# Exosomes: Biological Pharmaceutical Nanovectors for Theranostics

**DOI:** 10.3389/fbioe.2021.808614

**Published:** 2022-01-12

**Authors:** Shindu C. Thomas, Jin-Woo Kim, Giovanni M. Pauletti, Daniel J. Hassett, Nalinikanth Kotagiri

**Affiliations:** ^1^ Division of Pharmaceutical Sciences, James L. Winkle College of Pharmacy, University of Cincinnati, Cincinnati, OH, United States; ^2^ Department of Biological and Agricultural Engineering, Institute for Nanoscience and Engineering, University of Arkansas, Fayetteville, AR, United States; ^3^ St. Louis College of Pharmacy, University of Health Sciences and Pharmacy in St. Louis, St. Louis, MO, United States; ^4^ Department of Molecular Genetics, Biochemistry and Microbiology, University of Cincinnati College of Medicine, Cincinnati, OH, United States

**Keywords:** exosomes, outer membrane vesicles, theranostics, drug delivery, biodistribution, clinical trials

## Abstract

Exosomes are natural cell-derived nanovesicles of endocytic origin that enable cellular crosstalk by transferring encapsulated molecular cargos across biological barriers, thereby holding significantly complex implications in the etiology and progression of diverse disease states. Consequently, the development of exosomes-based nano-theranostic strategies has received immense consideration for advancing therapeutic interventions and disease prognosis. Their favorable biopharmaceutical properties make exosomes a unique nanoparticulate carrier for pharmaceutical drug delivery. This review provides an update on the contemporary strategies utilizing exosomes for theranostic applications in nanomedicine. In addition, we provide a synopsis of exosomal features and insights into strategic modifications that control *in vivo* biodistribution. We further discuss their opportunities, merits and pitfalls for cell/tissue targeted drug delivery in personalized nanotherapy.

## 1 Introduction

Over the past few decades, exosomes have received considerable attention for their purported role in modifying cellular functions. Exosomes are membrane-bound nanoscale vesicles formed initially as a specific population of intraluminal vesicles by the invagination of the late endosomes. These intraluminal vesicles of endosomal origin are then released into the extracellular milieu as a consequence of the fusion of late multivesicular endosomes with the plasma membrane, a biogenesis mechanism which distinguishes them from other classes of extracellular vesicles (Hessvik and Llorente, 2018; van Niel et al., 2018). Thus, the exosomal outer membrane consists of a phospholipid bilayer enriched with donor cell membrane-derived proteins and an inner aqueous core that inherits cytoplasmic biomolecules which include proteins, enzymes, mRNA, miRNA and metabolites. Exosomes have a size that lies between 20–120 nm and vary morphologically and structurally based on the parent cell (Colao et al., 2018). Exosomes subpopulations exhibit diverse shapes ranging from spherical to filamentous and elongated, and can also include distinct sub compartments (Zabeo et al., 2017). Multiple isolation strategies have been developed to isolate exosomes from wide variety of biological sources which include fluids, such as milk, blood and urine; and plant-derived products, such as fruits and vegetables. Extracellularly released exosomes can exhibit partial organ selectivity, which is because of its unique proteome and lipid composition, sharing significant similarity with the donor cell, subsequently affecting its cellular uptake (Hoshino et al., 2015; Sancho-Albero et al., 2019). Exosomes exploit multiple endocytic mechanisms to enter cells. Interestingly, exosomes can also overcome the species barrier to deliver biomolecules between cells of different origin (Zhou et al., 2017; Baldini et al., 2018; Chen et al., 2019; Lee, 2019; Schuh et al., 2019). Exosomes mediate biological functions and thus regulate vital physiological and pathological processes *in vivo* (Li et al., 2020; Wang et al., 2020; Laksono et al., 2021). Since exosomes are natural shuttles of endogenously produced bioactive biomolecules, they have been extensively used for active and passive delivery of synthetic biomolecules. Their endogenous nature and nanoparticulate characteristics also allow these nanocarriers to migrate across biological barriers and imparts them with excellent biocompatibility (Akuma et al., 2019; Elliott and He, 2021).

In this review, we discuss the current strategies utilizing exosomes for disease prognosis and as a potential bio-therapeutic vector. We further discuss bacterial outer membrane vesicles as a similar nanocarrier in drug delivery. Also, we discuss the biopharmaceutical properties of exosomes focusing specially on its *in vivo* biodistribution and the potential benefits of utilizing exosomes over other traditional nanocarriers for personalized theranostic nanomedicine. We conclude the article by identifying the challenges associated with developing exosome based nanotheranostic strategies and comment on future perspectives for its clinical translation and pharmaceutical adoption.

## 2 Exosome Isolation and Characterization

**FIGURE 1:**
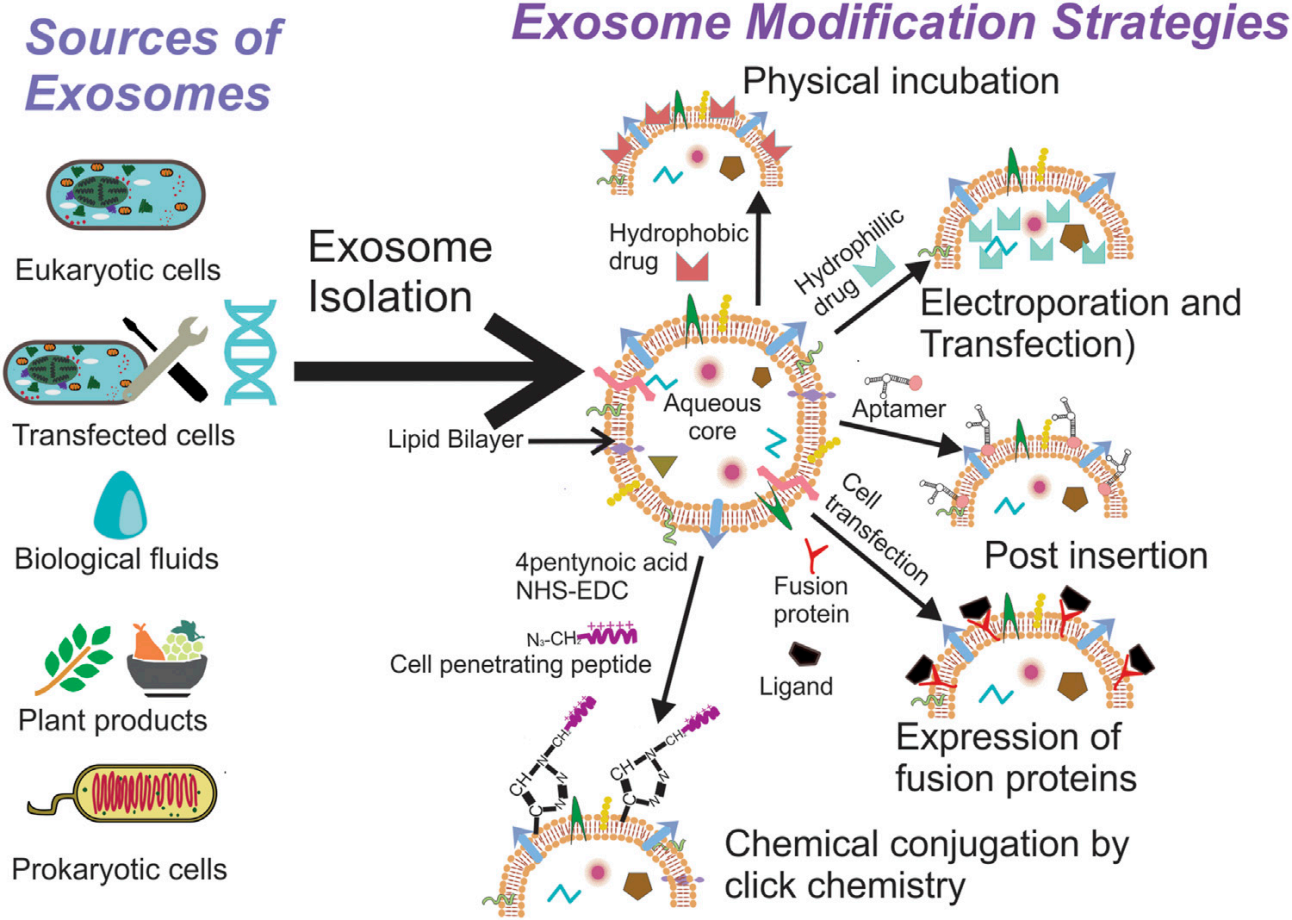
Exosomes can be isolated from a variety of sources, the selection of which depends on the intended application. Once isolated they can used directly for disease therapy or can be modified further to suit the intended application.

Exosomes have been isolated from a variety of cells and biological fluids (Figure 1), the choice of which depends upon the intended application. Exosomes from mammalian cells, such as mesenchymal stem cells (MSC) and dendritic cells (DC), have been used to prepare vaccines and direct therapeutic interventions. Tumor cell-derived exosomes (TEx) circulating in human plasma and from cultured cancer cells have been isolated to identify diagnostic biomarkers. Biological fluids, such as plasma and milk, contain exosomes secreted by a variety of cells and the exosome yield from such sources is typically high (for e.g., ∼300 mg of exosomes/liter of milk) (Munagala et al., 2016). Similarly, outer membrane vesicles (OMVs) have been isolated from bacterial cultures to explore their functionality and therapeutic potential *in vivo.* Depending on the biological source, the selection of an appropriate isolation method is critical for yield, purity and the processing costs. We summarize the different methods for exosome isolation in Table 1. The general methods to identify exosomes and analyze their functionality are described in Table 2 (Witwer et al., 2013; Thery et al., 2018). Once exosomes are isolated, it is necessary to store them under optimum conditions to maintain their morphology and structural integrity, to prevent contamination and aggregation, and to obtain maximum recovery for downstream processing. Storage conditions have been shown to affect the activity and stability of exosomes. Storing exosomes at 20°C or 4°C results in a significant decrease in particle number and biological activity, while −80°C storage is reported as the preferred condition for long-term storage with insignificant degradation or loss of biological activity for up to 6 months (Munagala et al., 2016; Cheng Y. et al., 2019; Wu et al., 2021b). Lyophilization with the addition of a cryoprotectant is an alternate method that allows exosomes to be stored at room temperature to up to 4 weeks without a significant loss in biological activity (Charoenviriyakul et al., 2018b; Trenkenschuh et al., 2021). Patented lyophilization methods have been developed that allow exosome storage for longer periods but under refrigerated conditions, allowing the commercialization and transport of exosome-based products (Sitar et al., 2015; Lim, 2019).

**TABLE 1:** General parameters and methods for characterizing exosomes (Witwer et al., 2013; Thery et al., 2018).

Parameter	General requirements	Conventional experimental technique used
Exosome quantification	Total protein content	BCA assay
	Total particle number	Nanoparticle tracking analysis, Resistive pulse sensing
	Total lipid content	Sulfophosphovanilin assay Fluorescence measurements of phospholipid dyes
		Total reflection fourier-transform infrared spectroscopy
Exosome purity	At least one comparative ratio including particle number, total protein and lipid content	Methods as discussed under exosome quantitation
Exosome markers	-Presence of 3 protein markers including transmembrane/lipid-bound (e.g., Tetraspannins, MHC, Integrins) and cytosolic proteins (e.g., ESCRT-I/II/III associated and accessory proteins)	Immunoblotting
	-One negative protein marker (e.g., Apolipoproteins)	Flow cytometry
		Confocal laser scanning microscopy (CLSM) using fluorescent antibodies
Exosome size and morphology	Two different but complementary techniques to study particle morphology, size and size distribution	Dynamic light scattering, Nanoparticle tracking analysis, Electron microscopy
		Atomic force microscopy, very Small-angle neutron scattering
Detecting surface topology of active components	To test the presence of at least one luminal or cytosolic cell component	Enzymatic and detergent based digestion of luminal and surface components
		CLSM using fluorescent antibodies
Exosome functionality	*Ex-vivo* dose response studies with appropriate positive and negative controls	Basic cell culture techniques and tests for measuring cellular responses

**TABLE 2:** A brief summary of conventional methods for isolating exosomes with their respective advantages and disadvantages (Witwer et al., 2013; Thery et al., 2018).

Isolation technique	Principle	Advantages	Disadvantages
Differential centrifugation	Density and size-based separation involving multiple centrifugation steps	Inexpensive processing, supports all sample volumes, high yield	Acceptable purity, time consuming run times, labor intensive multiple steps, expensive equipment’s
			large RCF values might affect exosome integrity, particle aggregation
Polymeric Isolation reagents	Alter exosome dispersibility with polymers such as PEG resulting in exosome precipitation	Easy and quick processing, supports all sample volumes, inexpensive equipment’s	Possible contamination with polymers and co-precipitation of additional proteins and cells, expensive isolation reagents
Exosome precipitation with salts	Precipitate exosomes by altering surface charge/zeta potential with salts such as sodium acetate	Easy and quick processing, supports all sample volumes, inexpensive equipment’s and reagents	Possible contamination due to co-precipitation of additional proteins and cells
Immunoaffinity isolation	Use exosome surface marker specific antibodies immobilized on a substrate to preferentially isolate exosomes	Excellent purity due to high specificity	Low yield, expensive reagents does not support large sample volumes, requires preprocessing to remove cells and debris, tumor heterogeneity affects repeatability and reproducibility
Size exclusion liquid chromatography	Size based isolation of exosomes	Excellent exosome integrity, purity, yield and method reproducibility	Moderately expensive, requires specialized equipment’s, time consuming, does not support large sample volumes
Ultrafiltration	Size based isolation of exosomes	Quick processing, good purity, portable, inexpensive equipment’s	Acceptable purity, moderate yield, does not support large sample volumes, exosome deformation due to applied force, column blockage leads to low elution efficiency
Microfluidic systems	Microchannels isolating exosomes by combining exosome specific antibodies and size-based enrichment	Quick processing, automated and portable, excellent purity	Does not support large volumes, requires expensive equipment’s, technical expertise in microfluidics required for process development

## 3 Therapeutic Applications

### 3.1 Unmodified Exosomes for Disease Therapy

Exosomes are bioactive and their direct administration has vital implication in modifying cellular responses. These nanoparticulate vectors carry multiple signaling and regulatory molecules that exert their biological functions via distinct molecular mechanisms. The curative potential of these molecules is currently being explored in clinical trials (Table 3) (ClinicalTrials.gov, 2021). Since exosomes have been isolated from different parent cells/sources, we discuss the most significant ones in this section.

**TABLE 3:** Clinical trials utilizing exosomes for therapeutic benefits and drug delivery (Clinicaltrials.gov).

Study title [NCT number]	Isolation source	Phase	Therapeutic condition	Status/Outcome
***Unmodified exosomes for therapeutic effect***				
Effect of plasma derived exosomes on cutaneous wound healing [NCT02565264]	Autologous plasma derived	Early phase 1	Wound healing (Ulcers)	Enrolment by invitation
Plant exosomes and patients diagnosed with polycystic ovary syndrome (PCOS) 17 [NCT03493984]	Ginger and Aloe	Not applicable	Polycystic ovary syndrome	Not yet recruiting
MSC-Exos promote healing of MHS [NCT03437759]	Mesenchymal stem cells	Early phase 1	Macular hole	Recruiting
Edible plant exosome ability to prevent oral mucositis associated with chemoradiation treatment of head and neck cancer [NCT01668849]	Grapes	Phase 1	Head and neck cancer	Active, not recruiting
			Oral mucositis	
Pilot immunotherapy trial for recurrent malignant gliomas [NCT01550523]	IGF-1R antisense oligodeoxynucleotide treated autologous glioma cells	Phase 1	Malignant brain glioma	Completed
Antisense 102: Pilot immunotherapy for newly diagnosed Malignant Glioma [NCT02507583]	IGF-1R antisense oligodeoxynucleotide treated autologous glioma cells	Phase 1	Malignant glioma neoplasms	Active, not recruiting
Extracellular vesicle infusion treatment for COVID-19 associated ARDS (EXIT-COVID19) [NCT04493242]	Bone marrow derived exosomes	Phase 2	COVID-19	Completed
A pilot clinical study on inhalation of mesenchymal stem cells exosomes treating severe novel coronavirus pneumonia [NCT04276987]	Allogenic adipose mesenchymal stem cells	Phase 1	COVID-19	Completed
Evaluation of safety and efficiency of method of exosome inhalation in SARS-CoV-2 associated Pneumonia. (COVID-19EXO) [NCT04491240]	Mesenchymal stromal cells-derived exosomes	Phase 1	COVID-19	Completed
		Phase 2		
The use of exosomes for the treatment of acute respiratory distress syndrome or novel coronavirus pneumonia caused by COVID-19 (ARDOXSO) [NCT04798716]	Mesenchymal stromal cells-derived exosomes	Phase 1	COVID-19	Not yet recruiting
		Phase 2		
Expanded access to Zofin^TM^ (Organicell^TM^ flow) for patients with COVID-19 [NCT04384445]	Human amniotic fluid derived exosomes in combination with other immunoregulatory agents	Phase 1	COVID-19	Recruiting
		Phase 2		
Expanded access protocol on bone marrow mesenchymal stem cell derived extracellular vesicle infusion treatment for patients with COVID-19 Associated ARDS [NCT04657458]	Bone marrow MSC	Phase 2	COVID-19	Completed
***Drug delivery***				
Study investigating the ability of plant exosomes to deliver curcumin to normal and colon cancer tissue [NCT01294072]	Fruit derived exosomes loaded with curcumin	Phase 1	Colon cancer	Active, not recruiting
Iexosomes in treating participants with metastatic pancreas cancer with KRASG12D mutation [NCT03608631]	Mesenchymal stromal cells-derived exosomes loaded with KRAS G12D siRNA	Phase 1	Metastatic pancreatic adenocarcinoma	Not yet recruiting
			Stage IV pancreatic cancer	
Allogenic mesenchymal stem cell derived exosome in patients with acute Ischemic stroke [NCT03384433]	Allogenic MSC exosome loaded with miR-124	Phase 1	Acute ischemic stroke	Not yet recruiting
		Phase 2		
Trial of a vaccination with tumor antigen-loaded dendritic cell-derived exosomes (CSET 1437) [NCT01159288]	Dendritic cells	Phase 2	Non-small cell lung cancer	Completed
Exosome-based nanoplatform for Ldlr mRNA delivery in FH (ENDFH) [NCT05043181]	Donor bone marrow derived MSCs with Ldlr mRNA	Phase 1	Familial hypercholesterolemia	Not yet recruiting
Evaluation of the safety of CD24-exosomes in patients with COVID-19 infection [NCT04747574]	Human embryonic kidney 293 cells engineered to overexpress CD24	Phase I	COVID-19 (preventing cytokine storm)	Recruiting
A phase II randomized, double-blind, placebo-controlled study to evaluate the safety and efficacy of exosomes overexpressing CD24 to prevent clinical deterioration in patients with moderate or severe COVID-19 infection [NCT04969172]	Human embryonic kidney 293 cells engineered to overexpress CD24	Phase 2	COVID-19 (preventing cytokine storm)	Active, not recruiting
Safety and efficacy of exosomes overexpressing CD24 in two doses for patients with moderate or severe COVID-19 [NCT04902183]	—	Phase 2	COVID-19 (preventing cytokine storm)	Recruiting
COVID-19 specific T cell derived exosomes (CSTC-Exo) [NCT04389385]	Allogenic T-cell activated *in vitro* with viral peptide fragments	Phase 1	COVID-19	Active, not recruiting

#### 3.1.1 Mesenchymal Stem Cell Derived Exosomes

Exosomes isolated from mesenchymal stem cells (MSCex) originating from different tissues have been studied extensively for their therapeutic benefits by exerting a robust immunomodulatory effect. Ma et al. reported adipose tissue derived MSCex to promote wound healing *via* the activation of Wnt/β-catenin signaling pathway to inhibit apoptosis and facilitate cell proliferation and migration (Ma T. et al., 2019). In a similar manner, Han et al. induced hypoxic stress in adipose tissue derived MSC and observed that the secreted exosomes were enriched with biomolecules, such as the vascular endothelial growth factor (VEGF), epidermal growth factor (EGF) and the fibroblast growth factor. These exosomes were shown to promote angiogenesis in fat grafting by regulating VEGF/VEGF-R signaling (Han et al., 2019). Encapsulated neural growth factors, such as glial cell-derived neurotrophic factor, fibroblast growth factor-1, brain-derived neurotrophic factor and the insulin-like growth factor-1 allowed them to promote nerve regeneration and repair (Bucan et al., 2019). Dalirfardouei et al. isolated exosomes from menstrual blood derived MSC and found them to be therapeutically active in hard-to-heal wounds. These exosomes suppressed inflammation by enhancing the M1 to M2 macrophage polarization and promoted wound healing by the upregulation of nuclear factor-κB (NF-κB) pathway in diabetic rat models. Parallel to adipose tissue derived MSCex, they promoted neoangiogenesis and enhanced the synthesis of collagen and elastin fibers (Dalirfardouei et al., 2019). A similar anti-inflammatory potential was also observed by Wei et al. for human placenta derived MSCex as they induced the preferential anti-osteogenic M2c macrophage polarization and contained biomolecules like VEGF and miRNAs belonging to the family of miRNA126 and miRNA145, enabling the regeneration of endothelium and vascular smooth muscle (Wei et al., 2019). Ma et al. tested human umbilical cord MSCex for treating peripheral nerve injury via their immunomodulatory potential. The exosomes promoted motor functions, regeneration of axons and overall functional recovery by regulating multiple cytokines such as interleukins (IL) 6, 1β and 10 (Ma Y. et al., 2019). Alternatively, bone marrow MSCex have also been demonstrated to promote tissue regeneration by multiple mechanisms, which include the suppression of pro-inflammatory interleukins, inhibiting tumor necrosis factor-α (TNFα) mediated collagenase activity and cyclooxegenase-2 upregulation, and the promotion of matrix proteoglycans and collagen production (Vonk et al., 2018). Consistent with these observations, Qi et al. further showed that bone marrow MSCex could inhibit chondrocyte apoptosis by regulating the P38 mitogen-activated protein kinases, protein kinase B or Akt and MAPK/ERK pathways in response to IL-1, which is a key mediator of articular cartilage degeneration in rheumatoid arthritis and osteoarthritis (Qi et al., 2019). MSCex thus induce a favorable microenvironment enhancing tissue regeneration by modulating inflammation, attenuating apoptosis and promoting cellular proliferation.

MSCex also exert a neuroprotective effect, which could be applied in reversing neurological damage and improving recovery rates. Cui et al. studied bone marrow MSCex and showed that they could significantly ameliorate cognitive functions. Exosomes decreased neuronal inflammation by modulating the signal transducer and activator of transcription 3 and NF-κB pathways and by increasing the expression of synaptic proteins, such as synapsin 1, to improve synaptic transmission (Cui et al., 2018). Similar observations have also been made for MSCex in animal models of brain injury and hemorrhagic shock (Williams et al., 2019). Apart from the neuroprotective effect of MSCex, their contents have also been shown to prevent organ damage. Li et al. showed that bone marrow MSCex contain anti-apoptotic miR-21-5p that conferred protection against oxidative stress-induced cell death and alleviated ischemic/perfusion related lung injury (Li J.w. et al., 2019). Further, encapsulated miR-21a-5p, miR-125b and miR-199a-5p were shown to downregulate proapoptotic genes (e.g., phosphatase and tensin homolog and Fas ligand synthesis), suppress endoplasmic reticulum stress that protects against renal ischemia/reperfusion injury and kidney failure, and asserting a cardioprotective effect (Luther et al., 2018; Xiao et al., 2018; Wang et al., 2019). Bone marrow MSCex have also been shown to protect against chronic liver inflammation in response to autoimmune hepatitis by transferring miR-223 to the hepatocytes and modulating cryopyrin (NLRP3) and caspase-1 dependent mechanisms (Chen et al., 2018). Similarly, human umbilical cord MSCex have also been shown to alleviate hyperglycemia in type II diabetes mellitus by inhibiting pancreatic β-cell apoptosis, promoting insulin sensitivity and increasing glucose uptake and metabolism in peripheral tissues (Sun et al., 2018b).

Exosomes secreted from MSC have inhibitory effects against neoplastic cells as well. Wu et al. demonstrated the inhibitory role of miR-126-3p containing bone marrow MSCex in pancreatic cancer. They reported the exosomes to be capable of inhibiting the proliferation and metastasis of pancreatic cancer cells by downregulating disintegrin and metalloproteinase 9, thus efficiently inhibiting tumor progression (Wu et al., 2019). Rosenberger et al. assessed the antitumor effects of menstrual MSCex in a hamster buccal pouch oral squamous carcinoma model. They observed that the exosomes could decrease VEGF production and inhibit angiogenesis in the tumor vasculature (Rosenberger et al., 2019). MSCex have also been reported to enhance the sensitivity of cancer cells towards radiotherapy (de Araujo Farias et al., 2018).

A vital pathophysiological hallmark of the highly contagious COVID-19 disease is the development of an acute respiratory distress syndrome that involves the development of pulmonary inflammation, lung fluid buildup, and pneumonia. Immunoregulatory agents such as lipids, transcriptional factors and proteins have been identified within circulatory plasma exosomes in clinical samples that are directly involved in the etiology and progression of COVID-19 infections (Azevedo et al., 2007; Song et al., 2020; Ahmed et al., 2021; Sur et al., 2021). The biological role of exosomes in cellular communication is now being evaluated to antagonize the severe inflammatory immune response, i.e., the cytokine storm. Bone marrow derived MSCex (ExoFlo™) in clinical trials has been shown to have immunomodulatory effects by downregulating the cytokine storm and bringing about an overall improvement in lung perfusion and oxygenation (Sengupta et al., 2020). Inducing MSC to secrete exosomes packed with regulatory factors can be a therapeutically superior option as demonstrated by Kaspi et al. (2021). “NurOwn™” exosomes obtained from bone marrow MSC stimulated *in vitro* to produce neurotropic and immunomodulatory factors, have been shown to reduce respiratory distress related pulmonary system damage *via* its immunoregulatory effect, increasing oxygenation levels and also improving the pulmonary architecture (Kaspi et al., 2021). Clearly, MSCex have the potential to translate as a clinically relevant therapeutic option. Various clinical trials are currently evaluating the safety and efficacy of MSCex in human subjects (Table 3).

The amniotic tissue fluid stem cell secretome packed inside amniotic fluid exosomes has an anti-inflammatory, immunomodulatory and tissue repair/regeneration potential similar to MSCex derived from other tissues. These exosomes enriched with growth factors and immunoregulatory proteins such as the transforming growth factor β (TGF-β), have been successfully employed in reducing oxidative damage, restoring cartilage strength and morphology in animal models of osteoarthritis and osteoporosis (Gatti et al., 2020a; Zavatti et al., 2020; O’Connell et al., 2021). Amniotic fluid derived exosomes exert a cardioprotective effect by modulating biological pathways, such as the PI3K-Akt signaling and Ca^2+^-dependent angiogenesis to promote cardiomyocyte proliferation and tissue repair in animal models of ischemic heart injury and myocardial infarction (Balbi et al., 2019; Takov et al., 2020). These vesicles have also been shown to improve neuronal functions in Alzheimer’s disease by amending the neuron morphology and viability and the extracellular levels of amyloid-β and phospho-tau protein levels. Gatti et al. demonstrated that the exosomes could reduce reactive oxygen species mediated oxidative stress by virtue of the naturally encapsulated enzymes such as superoxide dismutase 1 (Gatti et al., 2020b). Zofin™, a proprietary formulation containing amniotic fluid MSCex has been assessed in clinical trials to demonstrate significant therapeutic efficacy in improving respiratory functions in patients suffering from COVID-19 (Mitrani et al., 2021a; Mitrani et al., 2021b).

#### 3.1.2 Exosomes Isolated From Cardiosphere-Derived Cells

CDex possess regenerative and cardioprotective properties that preserve and protect the myocardial fiber microarchitecture (Nguyen et al., 2018). The cardioprotective activity is primarily attributed to the non-coding Y-RNAs that prime macrophages to secrete anti-inflammatory cytokine IL-10 (Cambier et al., 2017). Several CDex miRNAs belonging to miR-126, miR-130a and miR-210 family have been reported to exert a proangiogenic effect (Namazi et al., 2018). CDex possess anticancer properties as well. Grigorian-Shamagian et al. found CDex miRNAs belonging to the miR-146 family to inhibit tumor progression and vascularization, suggesting a protective effect on the heart from metastasizing cancer cells (Grigorian-Shamagian et al., 2017).

#### 3.1.3 Dendritic Cells Derived Exosomes

DEx have been explored as a viable alternative for anticancer immunotherapy. When dendritic cells are exposed to tumor derived antigens, they produce DEx surface expressing tumor antigens. Such DEx mimic the antigen presenting dendritic cells, stimulating an aggressive and specific immune response against the tumor cells by inducing the cytotoxic activity of CD8^+^ T cells (Zitvogel et al., 1998; Hegmans et al., 2005).

#### 3.1.4 Tumor Cell Derived Exosomes

TEx have been shown to possess tumor growth-promoting and immunomodulatory effects that might initiate an antitumor immune response (Hoshino et al., 2015). Benites et al. observed TEx isolated from patients to have an immunosuppressive effect, while TEx isolated from cultured cancer cells stimulated the immune system (Benites et al., 2019). Preconditioning or genetically engineering tumor cells is a promising approach to obtain immunostimulatory TEx. For instance, Guo et al. isolated exosomes from heat-stressed MC38 colon cancer cells and found them to be potent stimulators of the immune system by modulating dendritic cells to secret IL-6 and convert regulatory T-cells to helper T cells (Guo et al., 2018). Yang et al. transduced tumor cells with interferon regulatory factor 1 to produce TEx overexpressing IL-15Ra and the major histocompatibility complex-I, thereby increasing T-cell tumor infiltration (Yang et al., 2018).

#### 3.1.5 Plant Exosomes

Exosomes derived from plants have been shown to be absorbed into circulation and exert beneficial therapeutic effects (Liang et al., 2015). Mu et al. observed that exosomes isolated from different plant sources maintain intestinal homeostasis by modulating the production of anti-inflammatory cytokines and activating the Wnt signaling pathway (Mu et al., 2014). Exosomes isolated from lemons have been found to assert an anticancer effect by inducing TNF-related apoptosis-inducing ligand (TRAIL) mediated apoptotic activity in cancer cells (Raimondo et al., 2015). Ginger derived exosomes also have the potential to inhibit the progression of COVID-19 induced lung inflammation by virtue of their encapsulated miRNA (Teng et al., 2021).

### 3.2 Exosomes for Drug Delivery

Since exosomes are natural bio-shuttles of multiple bioactive molecules, their potential application as a drug delivery system (DDS) has triggered considerable interest over the past decade and therefore exosome-based DDS have eventually entered clinical trials (Table 3). Hence, innovative and diverse strategies to load exosomes with therapeutic molecules and engineer donor cells and exosomes have been devised to integrate unique properties within them (Figure 1). The simplest and the least invasive method to incorporate therapeutic molecules within exosomes is physical incubation, as reported by Sun et al. (2010) and Munagala et al. (2016). The research groups incorporated lipophilic anticancer molecules in exosomes derived from raw bovine milk and EL-4 mouse lymphoma cells, followed by testing them in tumor models *in vivo*. These exosomes could accumulate in the tumor tissue and promote significant tumor regression in comparison to the lipophilic anticancer drugs administered alone. Hydrophilic molecules, such as siRNA and miRNA, need to penetrate the lipid bilayer for efficient loading and hence alternative methods, such as electroporation and transfection, are preferable. Pommato et al. demonstrated that electroporation was more efficient in incorporating antitumor miR-31 and miR-451a inside plasma derived exosomes when compared with physical incubation and did not affect their integrity. Exosomes loaded with miRNA were able to silence target genes, promoting anti-apoptotic pathways in HEPG2 cancer cell lines (Pomatto et al., 2019). In a similar manner, Usman et al. electroporated exosomes isolated from red blood cells to load them with therapeutic RNA’s such as antisense oligonucleotides, Cas9 mRNA and guide RNA’s to target cancer cells in animal models of solid tumors and liquid cancers showcasing significant regression in tumor progression (Usman et al., 2018). Shokrollahi et al. loaded siRNA for HSP27 inhibition inside SH-SY5Y neuroblastoma cell-derived exosomes using lipofection reagents. The transfected exosomes were taken up by the cells to inhibit heat shock protein mediated neuronal differentiation (Shokrollahi et al., 2019). These studies show that exosomes have the ability to enter cells efficiently while protecting their cargo. Other methods, such as sonication, freeze-thaw cycles, extrusion through polycarbonate filters and incubation with membrane permeabilizers, have also been utilized to prepare drug loaded exosomes (Haney et al., 2015; Kim et al., 2016). The choice of a suitable method depends on the physicochemical properties of the agent being encapsulated. Coupled with multiple steps of processing and purification, these methods can affect exosomal integrity, which might subsequently result in differences between *in vitro* and *in vivo* behavior of exosomes. Alternatively, engineering the donor cell genome to pack biological molecules within exosomes is an exciting approach to manufacture exosomes enriched with therapeutic biomolecules *ex situ*. Li et al. used transduced γδ-T lymphocytes to overexpress miR-138, which has been established to have inhibitory effects on tumor cells via multiple mechanisms. Exosomes enriched with miR-138 were subsequently used to pre-immunize mice and found to have an immunomodulatory role effectively inhibiting tumor development *in vivo* (Li L. et al., 2019). Huang et al. transfected HEK293T cells with plasmids that encode for light sensitive protein interaction modules (i.e., CIBN–CYR2, MCP and MS2) and the endogenously produced miR-21 sponge. The system in response to light, accumulated miR-21 sponges on the plasma membrane that were ultimately packed into exosomes to be released *via* endocytosis (Huang et al., 2019).

A key challenge hampering current drug delivery systems is to achieve site-specific delivery with respect to an organ, a cell, and its sub-compartments. Exosome surface has thus been modified with multifunctional moieties to enhance their targeting potential by improving cell uptake, which is combined with the enhanced permeation and retention effect to reduce non-specific delivery. Jia et al. encapsulated superparamagnetic iron-oxide nanoparticles and curcumin inside macrophage derived exosomes to synergistically combine hyperthermia and imaging capabilities with an anticancer agent to inhibit cancer cells. To achieve cell specific delivery, a cell penetrating europilin-1-targeted peptide (RGERPPR, RGE) was conjugated to the exosome surface by click chemistry to obtain glioma cell targeting exosomes. The exosomes could penetrate the blood-brain barrier (BBB) easily and simultaneously provided excellent tumor imaging and inhibition capabilities (Jia et al., 2018). Similar to cell penetrating peptides, aptamers can also achieve efficient cell selectivity. Zou et al. synthesized diacyllipid-conjugated aptamers and inserted them into the lipid bilayer of exosomes *via* the post insertion method. The aptamer-modified, drug-loaded exosomes were tested on different cells and were found to show aptamer/cell-specific uptake (Zou et al., 2019). Pham et al. used enzyme-catalyzed reactions to covalently join receptor specific peptides with proteins on the exosomal surface *in vitro*, showcasing stable peptide conjugation with minimal invasiveness to the vesicles. They used Sortase A and OaAEP1 ligase to decorate the exosome surface with over 350 copies of EGFR specific nanobodies/exosome. The exosomes were shown to have a significantly higher propensity for tumor accumulation in comparison to unmodified particles. A higher tumor bioavailability also allowed the authors to reduce the drug dosage considerably (Pham et al., 2021). Exosome surface modifications can help in attaining cellular co-localization as shown by Cheng H. et al. (2019). They synthesized a multifunctional chimeric peptide comprising an alkyl C_16_ chain, a photosensitizer protoporphyrin IX for photodynamic therapy and a nuclear targeting cell penetrating peptide. The chimeric peptide was then inserted into the lipid bilayer of mice plasma exosomes. The chimeric peptide modified exosomes were capable of disrupting cell membrane integrity of murine mammary carcinoma (4T1) cells, escape lysosomal degradation and effectively co-localize in the nucleus. Light-guided activation of protoporphyrin IX generated reactive oxygen species in the nucleus that disrupted nuclear functioning and induced cell death. Also, on testing the system *in vivo*, it was able to inhibit tumor growth precisely with minimal systemic toxicity.

To achieve cell specific delivery, transfecting donor cells to incorporate targeting moieties on the surface of exosomes during their biogenesis was demonstrated by Alvarez-Erviti et al. (2011). They transduced dendritic cells to express Lamp2b protein fused with neuron targeted RVG peptide, consequently obtaining exosomes expressing the same proteins on their extracellular surface. Electroporation was used to load the exosomes with siRNA against the BACE1 gene, a therapeutic target for treating Alzheimer’s disease. Efficient neuronal targeting was achieved, which resulted in a strong gene knockdown in neurons, microglia, and oligodendrocytes. In a similar manner, Wang et al. (2018b) transduced bone marrow-derived stem cells to isolate exosomes bearing LAMP2b protein fused with the ischemic myocardium targeting peptide (CSTSMLKAC). The modified exosomes were internalized by hypoxia injured H9C2 cells, which were observed to be accumulate in ischemic heart tissues *in vivo*, enhancing the direct therapeutic effect of the exosomes by reducing inflammation and enhancing cardiac functions. For targeting exosomes towards leukemic cells, Huang et al. (2019) modified the surface of exosomes by the post-insertion method with AS1411 aptamer conjugated to cholesterol. The exosomes were effective in blocking miR-21 mediated functions and induced leukemic cell apoptosis. Transfection of donor cells also enables expression of specific proteins on the exosomal surface that could be later used for fusing and loading them with therapeutic or targeting moieties. De Bonito et al. used lentiviral vectors to express nef^mut^ anchoring protein on the surface of exosomes, which were then fused with the oncogenic HPV-E7 protein to produce immunogenic exosomes (Di Bonito et al., 2017). During testing *in vivo*, the immunogenic exosomes could induce antigen specific generation of cytotoxic T-lymphocytes that inhibited the development of TC-1 lung tumors.

### 3.3 Exosomes as Diagnostic Biomarkers for Diseases

Exosomes are found abundantly in biological fluids and have been implicated in different pathological and physiological processes. Analyzing their cargo and associated surface markers has become a vital tool for the diagnosis and predicting the prognosis of different diseases. Thus, exosome concentration, specific cargos and surface markers have been identified in patients that help them differentiate from healthy subjects. Exosomes isolated from biological fluids (e.g*.*, urine, saliva, plasma and cerebrospinal fluids) and surgically resected tumor tissues are being actively analyzed in different trials (Table 4). Based on source cell/tissue, they can be divided into two simple categories for further discussion, i.e., invasive (plasma exosomes) and non-invasive methods (urine and salivary exosomes).

**TABLE 4:** Clinical trials evaluating exosomes as a diagnostic biomarker (ClinicalTrials.gov, 2019).

Study title [NCT number]	Exosome source	Therapeutic condition	Status/Outcome
Combined diagnosis of CT and exosome in early lung cancer [NCT03542253]	Tumor cell derived exosomes (TEx) from lung cancer tissue	Lung cancer	Not yet recruiting
A study of circulating exosome proteomics in gallbladder carcinoma patients (EXOGBC001) [NCT03581435]	TEx from plasma and tumor tissue	Proteinosis	Recruiting
		Gallbladder carcinoma	
ncRNAs in exosomes of cholangiocarcinoma [NCT03102268]	TEx from plasma and tumor tissue	Cholangiocarcinoma	Recruiting
		Benign biliary stricture	
New biomarkers in pancreatic cancer using EXPEL concept (PANEXPEL) [NCT03791073]	TEx from pancreatic mass	Pancreatic Cancer	Recruiting
Analyses of exosomes in the cerebrospinal fluid for breast cancer patients with suspicion of leptomeningeal metastasis. [NCT03974204]	TEx from cerebrospinal fluid and plasma	Breast cancer	Not yet recruiting
		Leptomeningeal metastasis	
Exosome testing as a screening modality for human papillomavirus positive oropharyngeal squamous cell carcinoma [NCT02147418]	Oropharyngeal rinses	Oropharyngeal cancer	Recruiting
Clinical validation of a urinary exosome gene signature in men presenting for suspicion of prostate cancer [NCT02702856]	Urine	Prostate cancer	Completed
Major activation of NCC in graft urinary exosomes (MANGUE) [NCT03503461]	Urine	Kidney transplantation	Completed
		Hypertension	
A study of exosome proteomics and hemodynamics in sepsis [NCT03267160]	Plasma and urine	Hemodynamic instability	Active, not recruiting
		Autophagy	
LRRK2 and other novel exosome proteins in parkinson’s disease [NCT01860118]	Plasma and urine	Parkinson’s disease	Completed
Sepsis-damaged organs-double-markers identification of organ failure using fluorescent nanoparticle tracking analysis [NCT03222986]	Plasma and urine	Sepsis with organ failure	Recruiting
Pilot study with the aim to quantify a stress protein in the blood and in the urine for the monitoring and early diagnosis of malignant solid tumors [NCT02662621]	Plasma and urine	Cancer	Recruiting
Predicting prognosis and recurrence of thyroid cancer via new biomarkers, urinary exosomal thyroglobulin and calectin-3 [NCT03488134]	Urine	Thyroid cancer	Recruiting
Clinical evaluation of the “ExoDx Prostate IntelliScore” (EPI) [NCT03031418]	Urine	Prostate cancer	Recruiting
New biomarkers and difficult-to-treat hypertension [NCT03034265]	Plasma and urine	Hypertension	Completed
To Investigate the diagnostic accuracy of exosomal microRNA in predicting the aggressiveness of prostate cancer in Chinese patients [NCT03911999]	Urine	Prostate cancer	Recruiting
Multicenter, prospective study for urinary exosomal biomarkers of kidney allograft tubulointerstitial fibrosis (UFO) [NCT03870542]	Urine	Renal fibrosis	Not yet recruiting
		Kidney transplant failure	
Anaplastic thyroid cancer and follicular thyroid cancer-derived exosomal analysis *via* treatment of lovastatin and vildagliptin and pilot prognostic study *via* urine exosomal biological markers in thyroid cancer patients [NCT02862470]	Urine	Thyroid cancer	Active, not recruiting
Early detection of autoimmune thyroid heart disease *via* urinary exosomal proteins [NCT03984006]	Urine	Thyroid diseases	Not yet recruiting
		Heart failure	
Characterization of adult onset autoimmune diabetes (CIAO) [NCT03971955]	Plasma	Diabetes	Not yet recruiting
MicroRNA as prediction and/or prognostic markers of IRIS in TB-HIV co-infected patients (miRNA) [NCT03941210]	Plasma	HIV and tuberculosis infection	Recruiting
Detection of circulating biomarkers of immunogenic cell death (ICD) [NCT02921854]	Plasma	Non-small cell lung cancer	Active, not recruiting
Exosomes in rectal cancer [NCT03874559]	Plasma	Rectal cancer	Recruiting
Identification and characterization of predictive factors of onset of bone metastases in cancer patients (PreMetOn) [NCT03895216]	Plasma	Bone metastases	Recruiting
Non-coding RNA in the exosome of the epithelia ovarian cancer [NCT03738319]	Plasma	High grade serous ovarian carcinoma	Recruiting

#### 3.3.1 Plasma Exosomes

Exosomes secreted by the majority of cells in the human body ultimately enter the interstitial fluid and blood plasma, which together form the major part of the extracellular fluid. Hence, plasma exosomes originating from different cells are a treasure of biomarker nucleic acids and proteins. Exosomes have been shown to play an important role in tumor development and metastasis (Hoshino et al., 2015). An increase in exosome concentration in plasma has also been shown to be a potential prognostic marker for different cancers such as of the breast, pancreas, lung and esophagus (Matsumoto et al., 2016; Sharma et al., 2017). Plasma exosomes derived from cancer patients have been analyzed for several diagnostic biomarkers, especially miRNAs. miR-1246 and miR-21 have been found to be enriched within plasma exosomes isolated from patients suffering from esophageal squamous cell carcinoma and breast cancer (Takeshita et al., 2013; Tanaka et al., 2013; Hannafon et al., 2016). Zhu et al. compared non-coding transfer RNAs in plasma exosomes isolated from liver cancer patients with those of healthy controls, and tRNA-ValTAC-3, tRNA-GlyTCC- 5, tRNA-ValAAC-5 and tRNA-GluCTC-5 were found to be significantly elevated in these patients (Zhu et al., 2019). Glypican-1+ plasma exosomes enriched in miR-95-5p and miR-149 have been proposed to be a specific diagnostic biomarker in patients suffering from colorectal cancer (Li et al., 2017). Other surface markers, such as CD63, Caveolin 1, tyrosinase-related protein 2, integrin α4β1, HSP70, HSP90, EGFR VIII and survivin, have been associated with the progression and development of different melanomas, glioblastomas and prostate cancer (Lin et al., 2015). Besides cancer, plasma exosomes have been shown to carry vital information, which indicates the development of other disorders. Wang L. et al. (2018) isolated exosome from heart failure patients and correlated the regulation of different miRNAs with the progression of cardiac fibrosis. They observed that the downregulation of miR-425 and miR-744 promoted cardiac fibrosis by directly affecting the transforming growth factor β1 signaling. In a similar manner, Takahiro et al. showed that elevated levels of miRNA-1 and miRNA-133a in serum of patients with cardiovascular diseases indicated potential myocardial damage (Kuwabara et al., 2011). α-Synuclein aggregates have been detected in biological fluids, depicting the development of pathological conditions such as parkinson’s disease. Plasma exosomes containing α-synuclein have been shown to a characteristic feature in parkinson’s patients (Shi et al., 2014). A reduction in the expression of plasma exosome proteins, such as clusterin and apolipoprotein 1, has been suggested to be potential candidate biomarker for parkinson’s disease (Kitamura et al., 2018).

#### 3.3.2 Urinary Exosomes

Analogous to plasma exosomes, urinary exosomes are also potential diagnostic biomarker for cancers associated with the renal and the reproductive system. Srivastava et al. (2018) analyzed the alteration in the levels of different miRNAs present in urinary exosomes. They reported that the enrichment of hsa-miR-200c-3p within urinary exosomes was a diagnostic signature for the development of endometrial cancer. Zhan et al. (2018) showed that urinary exosomes enriched with the long non-coding RNA MALAT1 and prostate cancer associated transcript 1 were associated with poor recurrence-free survival in bladder cancer. McKiernan et al. (2016) established urinary exosome gene expression profiling assay as a biomarker for the development and prognosis of prostate cancer. They correlated this method with prostate-specific antigen (PSA) based detection to show that the assay could differentiate between benign, low grade and high-grade prostate cancers.

Besides cancer, urinary exosomes have been shown to contain biomarkers for diagnosing renal malfunctioning and related disorders. Transmembrane protein 2, aquaporin-2, polycystin-1 and polycystin-2 have all been shown to be altered in patients suffering from polycystic kidney disease (Pocsfalvi et al., 2015). Increased expression of nephrin, transient receptor potential cation channel subfamily C member 6, inverted formin-2 and phospholipase A2 receptor in urinary exosomes has been associated with impaired glomerular functioning (Hogan et al., 2014). Moon et al. (2011) carried out a proteomic study to identify potential biomarker candidates associated with diabetic nephropathy in urinary exosomes. Several candidates like aminopeptidase N, vasorin precursor, α-1-antitrypsin and ceruloplasmin were identified for diagnosing nephrotic syndrome and for defining the underlining pathophysiology. Elevated levels of miR-29c within urinary exosomes have been found to be a promising marker for the diagnosis of renal interstitial fibrosis. Since the renal system plays an important role in the absorption and elimination of metabolites from systemic circulation, they could be used for diagnosing disorders related to other distinct organs. For instance, the proteins, leucine-rich repeat kinase 2 and α-synuclein have been found to be overexpressed within urinary exosomes isolated from patients having parkinson’s disease (Ho et al., 2014).

#### 3.3.3 Salivary Exosomes

Salivary exosomes have been shown to carry important signatures for diagnosing cancers and other disorders related to the digestive and the respiratory tract. Lin et al. evaluated and compared exosomal mRNA isolated from esophageal squamous cell carcinoma cells *in vitro* and from *in vivo* xenograft models and found them to be enriched with exosomal chimeric GOLM1-NAA35 RNA (Lin et al., 2019). They reported that GOLM1-NAA35 RNA was also found within salivary exosomes isolated from esophageal squamous cell carcinoma patients in comparison to cancer free controls and thus alteration in its level could be used as a suitable marker to measure disease progression and patient responsiveness to chemotherapy. A similar study compared exosomal miRNA isolated from different head and neck squamous cancer cells to that of salivary exosomes from head and neck squamous cancer patients (Langevin et al., 2017). *miR-486-5p*, *miR-486-3p*, and *miR-10b-5p* were found to be substantially elevated in salivary exosomes isolated from head and neck squamous cancer patients. Sun et al. (2018a) performed an extensive proteomic study of human salivary exosomes to determine specific markers for lung cancer. Out of 785 proteins that they studied, BPI fold containing family A member 1, cornulin, mucin 5 subtype B and Ras GTPase-activating-like protein IQGAP1 were identified to be significantly dysregulated within salivary exosomes and were potential indicators of lung cancer development. Similarly, Machida et al. (2016) showed miR-1246 and miR-4644 as distinguishing salivary exosome markers for the diagnosis of pancreatobiliary tract cancer. Instead of sequencing nucleic acids, fourier-transform based infrared spectroscopy can also be used to show differences in the conformation of exosomal proteins, lipid and nucleic acids. Zlotogorski-Hurvitz et al. (2019) demonstrated the usefulness of this technique by studying the infrared spectral signatures of exosomes isolated from healthy individuals and patients suffering from oral cancer. The simple and indirect method could differentiate the exosomes and holds a promising potential for monitoring the development and progression of different cancers. Furthermore, Zheng et al. (2017) compared healthy controls with patients suffering from irritable bowel disease, such as Crohn’s disease, by testing salivary exosomes for differentiating biomarkers. Using liquid chromatography mass spectroscopy, they evaluated different proteins and found proteasome subunit alpha type 7 to be elevated in these patients. Hence, the biomarker can potentially replace colonoscopy for diagnosing irritable bowel disease. Similar to plasma exosomes, salivary exosomes enriched with differentiating markers have also been found to be potential indicators of central disorders such as parkinson’s disease (Cao et al., 2019).

## 4 Programming Exosomes for Modulating its Biodistribution *In Vivo*

Understanding the mechanism of interaction between exosomes, recipient cells and their *in vivo* fate is necessary for developing exosome based therapeutic systems. Exosomal surface proteins have been shown to interact with cells and play an important role in regulating their uptake. For instance, glycoproteins present on the surface of bovine milk exosomes modulate their cellular uptake via endocytosis in human and rat intestinal cells (Wolf et al., 2015). The degradation of O and N-surface glycans has also been shown to modulate exosomal cell uptake (Yamamoto et al., 2021). Exosomes employ multiple mechanisms of endocytosis to enter cells and the specific mechanism largely depends upon the recipient cell (Horibe et al., 2018). Apart from endocytosis, phagocytosis, micropinocytosis and fusion with the plasma membrane have also been shown to mediate their cellular uptake (McKelvey et al., 2015). Thus, the complex interaction between the exosomal surface proteins inherited from the donor cell and the receptor cell makes vital differences in its pharmacokinetic profile. Exosomes isolated from a variety of sources have been administered *in vivo* to characterize their pharmacokinetic behavior. Charoenviriyakul et al. isolated exosomes from RAW264 macrophages, B16BL6 melanoma cells, C2C12 myoblasts, NIH3T3 fibroblasts and MAEC aortic endothelial cell lines followed by labeling with a fluorescent fusion protein (i.e., gaussia luciferase-lactadherin) in order to evaluate their pharmacokinetic profile *in vivo* in healthy mice. The fluorescent protein tagged exosomes exhibited a very short half-life (i.e., 2–4 min) with the majority of the exosomes being rapidly redistributed to the liver after intravenous injection (i.v.), followed by clearance from systemic circulation (Charoenviriyakul et al., 2017). In a similar manner, Smyth et al., 2015 compared the pharmacokinetic behavior of fluorescently labeled TEx isolated from 4T1 and MCF-7 breast cancer and PC3 prostate cancer cells with hybrid liposomes formulated using exosomal and synthetic lipids (Smyth et al., 2015). When delivered intratumorally, TEx remained associated with tumor tissue to a significantly higher extent in comparison to liposomes. However, following i.v. injection, both TEx and hybrid liposomes showed comparable rapid clearance with fast liver and spleen accumulation, but with minimal tumor targeting. Thus, the unique exosomal lipid bilayer did not provide any significant advantage over liposomes after systemic administration.

Contrary to the above-mentioned conclusions, there are also reports in the literature that suggest exosomes isolated from alternative sources to have better circulation time and *in vivo* tumor accumulation potential. Millard et al. isolated MSCex from human umbilical vascular endothelial cells and encapsulated them with meta-tetra (hydroxyphenyl)chlorine (mTHPC), a photodynamic agent used in anticancer therapy (Millard et al., 2018). mTHPC-loaded exosomes were found to be stable *in vivo* and showed higher tumor accumulation in comparison to mTHPC alone and mTHPC loaded liposomes. Alternatively, Munagala et al. (2016) isolated raw bovine milk exosomes and loaded them with anticancer agents followed by *in vivo* administration orally and i.v. The exosomes circulated for a long period of time (up to 6 days) and accumulated in the tumor showcasing significant tumor regression. Moreover, after i.v. administration, the exosomes were observed to predominantly accumulate in the liver while significant fractions were also found in the lung, kidney, pancreas, spleen, ovaries, colon, and brain after oral administration. Similarly, Sun et al. (2010) also observed a long circulation profile for curcumin loaded EL-4 lymphoma cell derived exosomes with a significant redistribution to the liver and spleen after intraperitoneal (i.p.) and oral administration. On the other hand, orally administered HEK293 derived exosomes after peroral administration showed restricted distribution in the stomach and undetectable systemic absorption (Gupta et al., 2020). Thus, the biodistribution and elimination profile of exosomes seemingly depends upon the parent/donor cell. Wiklander et al. isolated exosomes from mouse dendritic cells, human bone marrow MSC, C2C12 myoblasts, HEK293T, B16-F10 melanoma and OLN-93 oligodendroglia cells and administered them i.v., i.p and subcutaneously (s.c.) in mice (Wiklander et al., 2015). Regardless of the source of exosomes, significant fractions were found to accumulate in the tumor tissue for each route of administration tested. However, there were significant quantitative differences in distribution when different routes of administration were compared. In contrast to i.v. injections, i.p. and s.c. injections resulted in significantly lower exosomal accumulation in liver and spleen whereas increased accumulation was observed in pancreas and gastrointestinal tract. Bioavailability of exosomes was much higher for i.p. and i.v. administrations in comparison to s.c. injection. Similar results were also obtained by Dhanu et al. for HEK293 cell derived exosomes tested *in vivo* (Gupta et al., 2020). The site of administration combined with the choice of donor cells should thus be selected carefully to ensure optimum exosome distribution to the target tissue. Since exosomes are composed of lipids and proteins similar to those present in the cell membrane, their ability to traverse the BBB after i.v. administration has also been investigated (Qu et al., 2018). Banks et al. tested exosomes isolated from cancerous and non-cancerous cell lines of different tissue origins for their ability to cross the BBB. They successfully demonstrated that each type of exosome tested could penetrate the BBB using transcytotic mechanisms, however, a variation in penetration between each type of exosome, as high as 10-fold, was also reported. Glycoproteins such as the wheatgerm agglutinin, innate immune status activation, BBB inflammation and receptor-ligand interactions such as with mannose-6-phosphate were found to modulate the penetration mechanism, however the effect of these factors were again found to be highly variable (Banks et al., 2020).

Exosomes inherit from their donor parent cells specific proteins and lipids that enhance their interaction with their donor cell tissue, as demonstrated by the identification of specific ligand-receptor interactions promoting cellular uptake. Thus, the unique composition of exosomes is the probable reason for the wide extent of variation in tissue distribution of exosomes isolated from various sources. Charoenviriyakul et al. (2018a) isolated exosomes from B16BL6 melanoma cells followed by treatment with proteinase K which resulted in the digestion of integrins present on the exosomal surface, thereby increasing its accumulation in the lungs. Consistent with these observations, Hoshino et al. also showed the capability of integrins in modulating exosome biodistribution. They compared tumor exosomes isolated from different mouse and human lung, liver and brain tumor cells cancer cells and demonstrated that integrins expressed on the exosomal surface are responsible for directing tissue specific accumulation and cell-specific uptake such as by the lung fibroblasts and epithelial cells, liver Kupffer cells and brain endothelial cells, thereby elucidating the role of Tex in creating a pre-metastatic niche (Hoshino et al., 2015). Specifically, integrin proteins present on the exosomal surface, such as α6β4 and α6β1 on lung TEx and αvβ5 on liver TEx, have been linked to the organ specific-metastatic behavior. Other integrins, such as CD47, have also been shown to help exosomes in evading phagocytosis by monocytes and macrophages (Kamerkar et al., 2017). Thus, the heterogeneity of surface proteins, which is inherited from the parent cell, imparts exosomes with a natural potential for preferential cell uptake (Sancho-Albero et al., 2019). Wiklander et al. (2015) studied the biodistribution potential of Dex and compared their biodistribution with exosomes isolated from cells of different tissue origin. A significantly higher percentage of DEx administered were found to accumulate in the spleen in comparison to exosomes originating from different cells. Their studies suggest that exosomes inherit a wide variety of surface receptors and extracellular matrix-binding proteins as their parent cell, which enables them to show parent cell-specific uptake to a significant extent. Tetraspanins, which are abundantly found on exosomal surface, have also been implicated in modulating exosome cell uptake. For instance, Rana et al. (2012) reported that only a specific population of exosomes expressing tetraspanin 8 and alpha 4 integrin complex are taken up selectively by endothelial and pancreatic cells by interacting with the CD54 ligand present on the cellular surface. This selectivity was confirmed for exosomes isolated from lymph node stromal cell lines after transfecting them to obtain exosomes expressing this complex and showing a similar uptake behavior.

The repertoire of bioactive components that an exosome inherits from its parent cell puts forwards an interesting hypothesis that exosomes are preferentially taken up by the parent cell, allowing their accumulation in the organ housing the parent cell. This hypothesis has been tested and the partiality of exosomes to be endocytosed by the originating cell in combination with the EPR effect has been applied for tumor tissue targeting. Thus, the affinity of ligands that are a component of the exosomal membrane proteome and lipids to specific receptors, and its physical attributes of size and surface charge allow it to move across biological barriers and entangle/trap in tightly packed tissues with poor drainage such as the tumor matrix. For e.g., exosomes isolated from the brain derived endothelial cells (hCMEC/D3 and bEnd.3 cells) have been shown to cross the BBB and target the brain after intravenous administration (Wu et al., 2021a; Kannavou et al., 2021). Plasma exosomes which are inherently derived from multiple tissues have also been used for targeted delivery towards the brain and improving drug bioavailability (Qi et al., 2020). Thus, exosomes have the typical ability of nanoparticulate drug delivery systems to target tumors *via* the EPR effect but it’s necessary to carefully screen and test them for predicting there *in vivo* circulation profile. If target localization is strictly desired, appropriate choice of the route of administration is necessary. For example, intranasal and/or intracerebral administration of exosomes could be utilized to circumvent other organs and maximize exosomal delivery to the brain (Perets et al., 2018). Biomimetic vesicles that are a hybrid of exosomes and exogenous lipids and polymers have also been shown to possess a superior pharmacokinetic profile (Wu et al., 2021a; Kannavou et al., 2021).

Alternative approaches involving surface modifications have also been adopted to modify the biodistribution profile of exosomes. Surface modifications with targeting ligands help in enhancing the preferential accumulation in tissues of interest. Wiklander et al. (2015) transfected HEK293T cells to produce exosomes expressing RVG, a ligand for the muscarinic receptor. The surface modified exosomes were able to accumulate in the brain tissue and significantly lower fractions were delivered to the other organs in comparison to unmodified HEK293T exosomes. Similar results were also observed for exosomes surface modified with folic acid ligand for enhancing accumulation in the tumor tissue (Munagala et al., 2016; Nguyen Cao et al., 2021). PEGylation of nanoparticles is a well-known method to increase the hydrophilicity of nanoparticle surfaces and prevent their elimination from systemic circulation by the reticuloendothelial system. Kooijmans et al. (2016) conjugated EGFR to phospholipid (DMPE)-PEG derivatives and introduced it into the exosome bilayer via the post-insertion mechanism, thereby achieving improved circulation time and cell-receptor specific targeting ability. This method did not affect exosome morphology, size distribution, or protein composition and increased the specific binding of exosomes to EGFR-overexpressing tumor cells. However, inconsistent results with PEGylation have also been reported and it might not always ensure an improvement in circulation for other types of exosomes (Kannavou et al., 2021).

Macrophage mediated pinocytosis plays a central role in exosomal clearance and, thus, strategically eliminating them *in vivo* has shown to enhance the exosome circulation time (Imai et al., 2015). Components of the exosomal surface have been investigated for their contribution towards this interaction. Royo et al. (2019) isolated exosomes from murine hepatocytes and treated them with neuraminidase to digest the sialic residues of exosomal surface glycoproteins. Removal of terminal sialic acid residues modified the hydrophilicity and altered the net surface charge of the exosomes, making it less negative. *In vivo*, a significantly higher fraction of neuraminidase treated exosomes were found to localize in lungs in comparison to the unmodified exosomes, which localized more in the spleen and liver. Saunderson et al. further demonstrated that sialic acid residues on exosomes interacted with CD169 molecules present on the surface of the macrophages, thereby resulting in their phagocytic uptake (Saunderson et al., 2014). Among the other surface molecules, galectin-5 has also been implicated to play a vital role in macrophage mediated uptake (Barres et al., 2010). Presence of phosphatidylserine on the extracellular side of recipient cells has been reported to be recognized by macrophages and initiate phagocytosis. Hence, phosphatidylserine has been investigated for its contribution to the pharmacokinetics of exosomes. Matsumoto et al. (2017) fabricated phosphatidylserine containing liposomes with a negative surface charge and administered them intravenously in mice, followed by the administration of B16BL6 melanoma cell exosomes. This unique strategy resulted in the accumulation of negatively charged liposomes in the liver, which further reduced the accumulation of exosomes in the liver and prolonged their systemic circulation time. Alternatively, increasing the magnitude of negative surface charge on exosomal surface has also been found to enhance macrophage mediated uptake (Kim and Mok, 2019). Watson et al. (2016) identified the scavenger receptor class A family of receptors that recognized negatively charged molecules on the surface of exosomes. They were able to reduce the accumulation of HEK293 cell derived exosomes in liver by blocking the receptor with negatively charged dextran sulphate.

## 5 Bacterial Extracellular Vesicles: Outer Membrane Vesicles-A Similar Facet

Analogous to eukaryotic exosomes, prokaryotic bacteria secrete nanosized membrane derived vesicles, known as the outer membrane vesicles (OMVs). OMVs are structurally similar to exosomes since they possess a hydrophilic central aqueous core surrounded by a lipophilic outer membrane, thus sharing similar physicochemical properties (Hessvik and Llorente, 2018; Toyofuku et al., 2019). These overlapping features allow them to be isolated, identified and characterized using the same methods as applicable for exosomes. OMVs like exosomes have a membrane structure similar to their donor bacterial cell, however, their mechanism of biogenesis is different. Exosomes are products of endosomal origin while OMVs are released due to the blebbing of the bacterial outer membrane and explosive cell lysis (Hessvik and Llorente, 2018; Toyofuku et al., 2019). OMVs in comparison to exosomes are bigger with their diameter lying anywhere between 20 and 400 nm. OMVs akin to exosomes carry cellular components, such as DNA, mRNA and proteins, that have the capability of modulating cellular actions and also act as secretory vehicles for bacterial products (Jagannadham and Chattopadhyay, 2015). Thus, depending on the donor cell and its composition, OMVs can be pathogenetic or nonpathogenic in nature. For instance, OMVs containing immunogenic molecular cargo can stimulate host innate and adaptive immunity while intestinal commensal bacteria derived OMVs have been shown to maintain intestinal homeostasis (Gilmore et al., 2021).

The easy programmability of bacteria and the many advancements in synthetic biology have enabled the development of OMVs packed with therapeutic recombinant proteins. To increase OMV yield, hypervesiculating strains can be developed by deleting or mutating genes such as the *nlpI*, *tolR*, and the *mlaE* gene or by overexpressing membrane proteases such as OmpT (Premjani et al., 2014; Chen et al., 2016; Ojima et al., 2020; Reimer et al., 2021). The addition of tris-glycine to the bacterial culture media has also been shown to increase OMV production *in vitro* (Hirayama and Nakao, 2020). OMVs have been employed *via* various routes of administration to deliver recombinant proteins and other xenobiotics, thereby inducing biological responses. Vaccination using OMVs containing foreign antigenic peptides has been reported to induce a robust antigen-specific immune response. Thus, OMV based vaccines can function in a manner similar to DEx and TEx to confer protection against pathogenic infections. For example, immunization with unmodified OMVs derived from the melioidosis causing gram-negative bacterium, *Burkholderia pseudomallei*, provided protection with no toxicity similar to a live attenuated vaccine by inducing an immune response via the upregulation of IgG, CD4^+^ and CD8^+^ T cells (Baker et al., 2021). In a similar manner, Li et al. (2018) vaccinated mice with OMVs isolated from *Trypanosoma gondii* and showed enhanced humoral and cellular immune responses against subsequent infections with prolonged survival time. OMVs isolated from other pathogenic bacterial strains, such as *Staphylococcus aureus*, *Salmonella typhimurium*, *Salmonella enteritidis* and *Brucella abortus* species, have also been demonstrated to be potential vaccine candidates (Wang et al., 2018c; Maiti et al., 2021; Solanki et al., 2021). Coakely et al. showed that OMVs isolated from *Heligmosomoides polygyrus* induced serum antibodies (i.e., IgG1, IgM and IgA) in mice and generated a strong immune resistance on subsequent exposure to the helminth (Coakley et al., 2017). Alternatively, OMVs can also be used as carriers of foreign antigens for immunization as demonstrated by Carvalho et al. They engineered the commensal gram-negative bacterium *Bacteroides thetaiotaomicron* to obtain OMVs packaged with foreign antigens of viral (Influenza virus A) and bacterial origin (*Salmonella enterica* ser. Typhimurium). The OMVs on intranasal and oral administration elicited a strong antigen-specific immune response systemically, especially in the mucosal tissues. OMVs packaged with a therapeutic protein, keratinocyte growth factor 2 were also tested for its efficacy in repairing intestinal tissue in mouse models of ulcerative colitis. When the OMVs were administered orally and intranasally, they observed reduced disease severity and enhanced intestinal tissue repair (Carvalho et al., 2019). Thus, OMVs can stably deliver therapeutic proteins orally, intravenously, and intranasally. Mammalian skin is home to a diverse population of commensal bacteria, hence bacterial OMVs can also be used for therapeutic applications transdermally as demonstrated by Gu et al. The research group isolated OMVs from *Escherichia coli*, loaded them with indocyanine green, followed by surface modification with melanoma cell targeting cell penetrating peptides. OMVs were demonstrated to penetrate the skin to a depth of at least 400 µm and interact with the primary melanoma tissue effectively to bring about an anticancer photothermal response upon near infrared irradiation (Peng et al., 2020). OMVs are hence equally versatile in comparison to exosomes as a carrier. Also, the ease of carrying out bacterial genome manipulations is a notable advantage over exosomes, enabling them to be loaded with recombinant proteins during biogenesis.

Non-pathogenic bacterial strains of *E. coli*, such as DH5alpha and BL21, have been used to manufacture OMVs displaying foreign antigens, therefore avoiding pathogenic bacterial components, and providing a safer alternative source for the production of OMVs. Watkins et al. modified a commercial strain of *E. coli*, (ClearColi) into a hypervesiculating stain, followed by plasmid bioengineering to express an antigen against the influenza A virus (matrix 2 protein) (Watkins et al., 2017). The OMV vaccinated mice displayed 100% survival after a lethal challenge with the Influenza virus. Additionally, the OMV based vaccine conferred protection against the H1N1 virus, implying the development of cross-species immunity. Similarly, Wang et al. modified the *E. coli* DH5alpha strain to express the tumoral antigen, human papillomavirus type 16 early protein E7 (Wang et al., 2017). The OMVs were tested in mice with TC1 lung cancer xenografts and found to induce an antigen-specific immune response, showcasing the upregulation of interferon-γ and significantly suppressing tumor progression. *E. coli* strains have also been modified to express peptides with affinity towards tagged proteins, making it possible to decorate the OMV surface with targeting moieties post-isolation. Cheng et al. (2021) utilized OMVs isolated from the *E. coli* Rosetta (DE3) strain to formulate an antitumor vaccine capable of displaying multiple tumoral antigens. They utilized the “plug-and-display” system to display “protein catchers” on the OMVs surface for post-isolation conjugation with tagged antigens. Through this technique, the OMVs were decorated with the neoantigen peptide ADPGK and OVA, which belong to the MC38 colon and B16-OVA melanoma tumor cells respectively and were able to elicit a synergistic antitumor response against the MC38 colon cancer and metastatic B16-OVA melanoma xenografts. This study showcased an innovative method for developing personalized tumor vaccines by simultaneously displaying multiple heterogenous antigens on the isolated OMVs. OMVs also have the potential to accumulate in tumors via the enhanced permeation and retention effect and induce an antitumor response by upregulating the production of antitumor cytokines CXCL10 and interferon-γ due to their intrinsic composition (Kim et al., 2017). Numerous studies have shown that OMVs isolated from bacteria can be further modified to be used as an effective nanoparticle drug delivery system for targeting tumors (Thomas et al., 2021). Gujrati et al. modified a mutant non-toxic *E. coli* K12 strain to display human epithelial growth factor ligand on OMV surface and loaded the OMVs with siRNA via electroporation for gene silencing and inhibiting the synthesis of kinesin spindle protein. OMVs were injected intravenously and were found to be effective in suppressing subcutaneous breast tumors. A simple protocol for drug loading, such as physical incubation, can also be used to load OMVs with chemotherapeutic agents as demsotrated by Kuerban et al. (2020). They prepared doxorubicin loaded OMVs *via* physical incubation and showed the accumulation of OMVs due to the EPR effect in tumor models *in vivo*. The OMVs brought about a significant regression in tumor growth rate and improved the pharmacokinetic profile of doxorubicin by prolonging its circulation. Hence, bacterial OMVs are truly versatile and hold a huge potential for drug delivery applications. They could prove to be the perfect alterative in overcoming the formulation related challenges associated with the conventional nanoparticulate systems.

## 6 Comparing Exosomes to Other Nano-Pharmaceuticals

At present, various US-FDA approved nano-formulations based on liposomes, inorganic nanoparticles, protein-drug nanocomposites, polymeric nanoparticles and micelles are used for theranostic applications (Bobo et al., 2016). Biocompatibility of these nanoparticles has been extensively studied and their toxicity profile has been well characterized. Metallic nanoparticles, for example, accumulate in organs, creating a homeostatic imbalance and lead to DNA damage, oxidative stress and inflammation (Sengul and Asmatulu, 2020). Liposomes are unilamellar vesicles similar to exosomes, which are often known to leak their encapsulated contents on interacting with blood components. Aggregation of liposomes *in vivo*, toxicity and immunogenicity because of their composition and charge further restricts their applications in the development of personalized medicine (Maja et al., 2020). Moreover, cell specific action of the current nanocarriers is an unresolved conundrum in developing strategies for precision medicine (Sharma et al., 2012). Furthermore, it has been reported that nanoparticles interact with various biological components *in vivo*, which are absorbed onto the nanoparticle-biological medium interface, commonly known as the protein corona. Formation of the protein corona layer changes nanoparticle surface characteristics drastically resulting in a major deviation in therapeutic efficacy (Rezaei et al., 2019). In comparison, exosomes can inherently circumvent some of these limitations. The exosome bilayer is a complex mixture of lipids, decorated with numerous distinct proteins, such as tetraspanins and integrins. As discussed previously, the exosomal bilayer has a composition similar to that of a cell membrane and this unique surface composition immensely affects its biodistribution. The *in vivo* formation of protein corona is also reported on the exosomal surface (Palviainen et al., 2020; Varga et al., 2020). Hence, making the right selection of an isolation source and the route of administration is crucial for preventing immunogenicity and rapid clearance. For instance, Munagala et al. (2016) used exosomes derived from raw bovine milk, which showed no significant short-term and long-term immunogenicity when tested *in vivo*. Fluorescently labelled bovine milk exosomes circulated *in vivo* for a long period of time and were able to inhibit tumor growth in xenograft tumor models. Membrane extruded and reformed cell membrane vesicles prepared *in vitro* in excellent yields have been shown to be exosome biomimetic with a long-circulating nature (Wu et al., 2021a). Unmodified and drug loaded MSCex and plant derived exosomes have been tested in clinical trials for safety and efficacy. Altering the surface composition with targeting ligands has shown to bring about considerable improvement in the biodistribution profile and cell/receptor targeted potential of exosomes (Nguyen Cao et al., 2021). Preparing hybrid and/or biomimetic exosomes can further help in developing attributes enhancing its penetration and target localization.

Exosomes are cellular products manufactured by the generation of intraluminal vesicles in the late endosomes during endocytosis (Hessvik and Llorente, 2018). Thus, the biogenicity of exosomes needs to be discussed as a disadvantage as well. Liposomes and other nanoparticles are synthesized using methods that allow scaling up and fine tuning of their physicochemical properties, such as size and yield. Since exosomes are isolated from living cells, bioengineering these cells is the only option to alter them at the source, which might not always be technically feasible. Moreover, since they are isolated from biological sources, their isolation process needs to be extensively optimized to remove contaminants, such as bigger extracellular vesicles, cell debris and proteins, and to maintain batch-to-batch homogeneity with acceptable yields. Even though lipophilic and hydrophilic agents can be encapsulated within exosomes, the efficiency of drug loading is another factor limiting its application. These challenges need to be addressed before fully realizing their potential for practical applications.

## 7 Conclusions and Outlook

**FIGURE 2:**
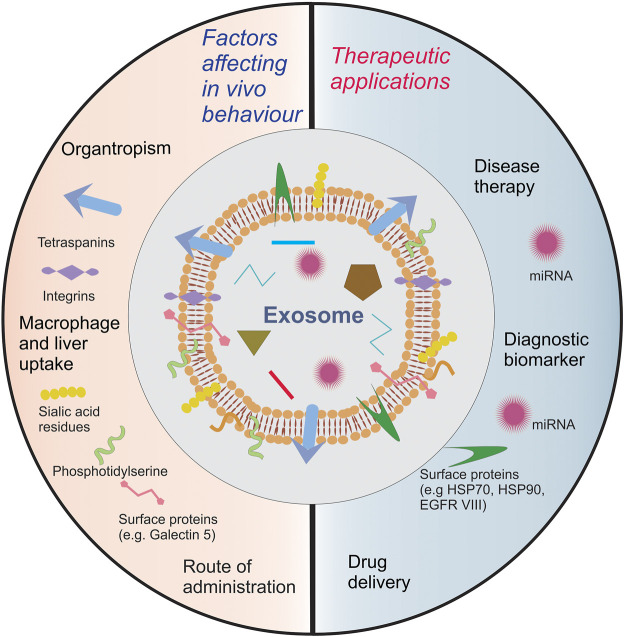
A summary of exosome biomolecules affecting exosome pharmacokinetics and their role in therapy.

Exosomes have been explored to define their role in the development of different pathological and physiological processes. Different methods for isolating exosomes have been standardized and the minimum requirements for exosome characterization have been well established. At the beginning of this review, we provided the reader with a brief update on these methods and the minimum requirements for exosome characterization. For successful clinical translation, protocols need to be developed, standardized, and streamlined to facilitate quick, reproducible, and inexpensive isolation of exosomes. We further discussed in detail the therapeutic applications of exosomes as an excellent natural nanocarrier of signaling biomolecules that exert remedial effects by directly acting on cells or as a diagnostic biomarker that represents disease progression and as a nanocarrier for drug delivery. Figure 2 summarizes the influence of these biomolecules on exosome pharmacokinetics and their applications in therapy. Since exosomes carry multiple signaling molecules, an important question that needs to be raised is of its safety and immunogenicity. These concerns can be primarily traced back to the parent cell/tissue. Biocompatibility, biodistribution and immunogenicity studies need to be conducted to identify potential safety concerns even though the biological origin of exosomes helps in minimizing them. Such safety studies are essential if the donor parent cell is a diseased cell, such as for tumor exosomes, which have been shown to support oncogenesis and tumor metastasis. Further, using autologous or allogenic exosomes might be an effective alternative to resolve unpredictable immunogenic concerns. Additionally, the biodistribution profile of exosomes can vary from source to source and it’s vital to screen them carefully before developing cell targeted systems for drug delivery. Promising solutions for enhancing their distribution are available and discussed in this review. However, additional technological advancements need to be made for achieving efficient selective cell targeting and enhancing the loading of exosomes. Engineering cells to load exosomes with biomolecules, expressing targeting moieties and enhancing exosome production, is an exciting but complicated approach. Irrespective of the technology used, maintaining batch-to-batch uniformity with respect to physicochemical and biological properties will be an uphill task for a formulation scientist. For successful commercialization of exosome based theranostic products, cost-effective methods for isolation and storage are still required. For instance, optimized lyophilization protocols have been developed by commercial manufacturers to make exosome products with longer shelf life, which might not always be feasible for laboratory scale research. Intensive investigations are also needed on other aspects, such as the non-specific biological interactions of exosomes *in vivo*.
